# Causes of Neonatal Deaths in a Rural Subdistrict of Bangladesh: Implications for Intervention

**DOI:** 10.3329/jhpn.v28i4.6044

**Published:** 2010-08

**Authors:** Hafizur Rahman Chowdhury, Sandra Thompson, Mohammed Ali, Nurul Alam, Md. Yunus, Peter Kim Streatfield

**Affiliations:** ^1^ Centre for International Health, Curtin University of Technology, GPO Box U1987, Perth, Australia; ^2^ ICDDR,B, GPO Box 128, Dhaka 1000, Bangladesh

**Keywords:** Causes of death, Interventions, Neonatal mortality, Verbal autopsy, Bangladesh

## Abstract

The study assessed the timing and causes of neonatal deaths in a rural area of Bangladesh. A population-based demographic surveillance system, run by the International Centre for Diarrhoeal Disease Research, Bangladesh, recorded livebirths and neonatal deaths during 2003-2004 among a population of 224,000 living in Matlab, a rural subdistrict of eastern Bangladesh. Deaths were investigated using the INDEPTH/World Health Organization verbal autopsy. Three physicians independently reviewed data from verbal autopsy interview to assign the cause of death. There were 11,291 livebirths and 365 neonatal deaths during the two-year period. The neonatal mortality rate was 32.3 per 1,000 livebirths. Thirty-seven percent of the neonatal deaths occurred within 24 hours, 76% within 0-3 days, 84% within 0-7 days, and the remaining 16% within 8-28 days. Birth asphyxia (45%), prematurity/low birthweight (15%), sepsis/meningitis (12%), respiratory distress syndrome (7%), and pneumonia (6%) were the major direct causes of death. Birth asphyxia (52.8%) was the single largest category of cause of death in the early neonatal period while meningitis/sepsis (48.3%) was the single largest category in the late neonatal period. The high proportion of deaths during the early neonatal period and the far-higher proportion of neonatal deaths caused by birth asphyxia compared to the global average (45% vs 23-29%) indicate the lack of skilled birth attendance and newborn care for the large majority of births that occur in the home in rural Bangladesh. Resuscitation of newborns and management of low-birthweight/premature babies need to be at the core of neonatal interventional packages in rural Bangladesh.

## INTRODUCTION

Deaths of newborns within 28 days of birth are a major barrier to improving the survival of children aged less than five years (under-five children) in developing countries. Neonatal deaths now account for more than two-thirds of all deaths in the first year of life and for about half of all deaths in under-five children (1,2). Bangladesh has a neonatal mortality rate of 41 per 1,000 livebirths, and neonatal deaths account for about half of deaths of under-five children (3). Therefore, appropriate interventions are crucial for improving the health of under-five children in Bangladesh and to help achieve the global target of reducing under-five mortality by two-thirds. Information on the timing and causes of neonatal deaths can help direct appropriate interventions.

According to the World Health Organization (WHO), preterm birth accounts for 30% of global neonatal deaths, sepsis or pneumonia for 27%, birth asphyxia for 23%, congenital abnormality for 6%, neonatal tetanus for 4%, diarrhoea for 3%, and other causes for 7% of all neonatal deaths (2,4,5). However, these estimates are based on limited datasets as most births and neonatal deaths occur in the home or outside formal health settings in developing countries (4,5). In this regard, verbal autopsy (VA) can be an appropriate and cost-effective tool as it uses a retrospective interview of family members about the circumstances of a death to ascertain the cause of death. The tool has been used successfully in many developing countries for generating reliable epidemiological data on mortality (6,7).

This paper describes the results of a VA study, particularly the direct cause of death and its timing, carried out on all neonatal deaths during 2003-2004 in a rural subdistrict of Bangladesh.

## MATERIALS AND METHODS

### Study design and population

The study was carried out in Matlab, a rural subdistrict in eastern Bangladesh, where the International Centre for Diarrhoeal Disease Research, Bangladesh (ICDDR,B) has been maintaining a longitudinal health and demographic surveillance system (HDSS) on ∼224,000 people living in the area (8). Approximately half of the surveillance population (n=112,000) receives a range of maternal and child-health and family-planning intervention services from ICDDR,B, and the remaining half receives standard government services (8). The demographic surveillance system is maintained at the peripheral level by the Community Health Research Workers (CHRWs) who make monthly visits to households to collect information on demographic events, such as birth, death, migration, abortion, marriage, divorce, etc., using pre-coded coloured forms to record the events. Living in the village and having a limited population to survey, these CHRWs are very unlikely to miss any vital events. These data are collated and maintained in the HDSS databases (8). The CHRWs collect data from both ICDDR,B and government service areas. This study investigated and analyzed all neonatal deaths in 2003 and 2004 in both intervention and comparison areas of the HDSS. The total number of neonatal deaths was 365.

### Verbal autopsy

The questionnaire was developed by a VA working group of the International Network of Field Sites with Continuous Demographic Evaluation of Populations and their Health in developing countries INDEPTH (http://www.indepth-network.org) and is closely based on the WHO VA questionnaire. For use in the Matlab HDSS, the questionnaire was adapted to local customs and culture and was translated into Bangla by a VA team at the Matlab HDSS before its piloting and implementation in the HDSS. The questionnaire included space for an open-ended description of deaths and a closed-ended component on pregnancy, child birth, and common illnesses of neonatal deaths. A structured one-page questionnaire was also included for collecting information on healthcare-seeking during the fatal illness episode.

### Collection of data

The CHRWs recorded any deaths in their assigned territory of households on a registration slip (date of death, identification number, etc.) during their monthly house-to-house visits, which was then sent to the block supervisor. These were then uploaded on the HDSS records. An interviewer trained in VA and with at least 10 years of schooling made a field-visit 2-6 weeks after the date of death to conduct the VA interview. After obtaining informed verbal consent, the interviewer conducted the interview in Bangla, the local language. Generally, the mother was the primary respondent but sometimes other family members were allowed to supplement the interview. Descriptive statements were recorded in the open part of the questionnaire in Bangla, preserving local idioms and refraining from any alteration or translation. Interviews generally lasted for 40-60 minutes depending on the history of illness and the emotional state of the caretakers.

### Assignment of cause of death

Three physicians independently reviewed all records of deaths to assign a direct cause of death and an originating/underlying cause of death when possible. The physicians worked at the ICDDR,B hospital in Matlab and were aware of the local disease profile of the population. They were also knowledgeable about the integrated management of childhood illness (IMCI) guidelines of the WHO (9). All physicians were given a brief orientation about the VA questionnaire and their responsibilities to allocate a cause of death. Their role was to review the completed VA questionnaire to assign a direct cause of death and an originating cause of death. They used a three-digit code for each cause of death from the list of codes of the tenth revision of International Statistical Classification of Disease, Injuries and Causes of Death (ICD-10) (10). An agreement of at least two physicians on a direct cause of death was required to assign a cause of death.

### Analysis of data

All data were entered via a Visual FoxPro data-entry screen into the Oracle database. For reporting purposes, prematurity and low birthweight were combined into one category, sepsis and meningitis into another category, and all congenital anomalies as one category. Deaths and causes of death were compared by sex of newborn, and the time period of death using χ^2^ test or Fisher's Exact test when appropriate. Data were analyzed using the Stata software (version 9) (11).

### Ethical approval

The Human Research Ethics Committee of the Curtin University of Technology and the Ethical Review Committee of the ICDDR,B approved the study. Informed verbal consent, confidentiality, and anonymity were ensured for all interviewees.

## RESULTS

### Early and late neonatal mortality rates by service area

The overall rate of neonatal morality in Matlab was 32.3 per 1,000 livebirths, with 30.6 in the ICDDR,B service area and 34.1 in the government service area. Table 1 shows that the rate of early (0-7 days) neonatal morality was 28.6 per 1,000 livebirths in the government service area, which was 11% higher than that in the ICDDR,B service area (25.8 per 1,000 livebirths). The rate of late (8-28 days) neonatal mortali ty was 5.5 per 1,000 livebirths in the government area, about 15% higher than that in the ICDDR,B area (4.8 per 1,000 livebirths). Overall, early neonatal deaths (first seven days of life) comprised 84% of all neonatal deaths.

**Table 1.** Neonatal mortality rate according to timing of death and service areas

Timing of death	ICDDR,B area (livebirth=5,659)	Government area (livebirth=5,632)	Both areas (livebirth=11,291)
Early (0-7 days) neonatal mortality rate*	25.8	28.6	27.2
Late (8-28 days) neonatal mortality rate	4.8	5.5	5.1
Total	30.6	34.1	32.3

The overall difference between areas is insignificant;

χ^2^ (1)=1.12; p=0.29;

*Neonatal mortality rate expressed per 1,000 livebirths

### Plurality of births

Most (82%) deceased neonates were singleton births while twins comprised 16% of the deaths, and one set of triplets accounted for 2%. Normally, about 1% of pregnancies result in a multiple birth. Plurality of birth was not significantly different between the ICDDR,B and the government service area.

### Age at neonatal death

Of the 365 neonatal deaths, 136 (37.3%) occurred on the day of birth, 279 (76.4%) within the first three days, and 307 (84.1%) within the first week of life (Fig.). Twenty-six (7.1%) of the deaths occurred during the second week, and the remaining 32 (8.8%) occurred in the third and the fourth week of life. Thus, more than four-fifths of the neonatal deaths occurred in the first week, with a large proportion on the day of birth.

**Fig.**
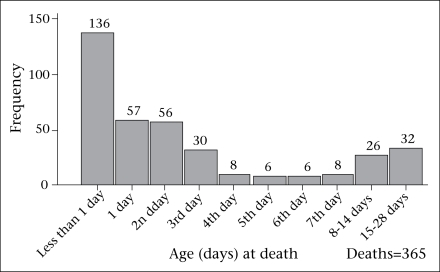
Age at death

### Cause of death

Birth asphyxia (44.9%), prematurity/low birthweight (15.1%), sepsis/meningitis (12.3%), respiratory distress syndrome (RDS) (6.9%), and pneumonia (5.5%) were the top five causes of death (Table 2). These five causes accounted for 85% of the cases. The other causes included hypothermia, birth injury, sudden infant death, and congenital anomalies. Around 7% of the cases were classified as undetermined as there was no agreement between any of the physicians on the cause assigned. In 1.9% of the cases, the physicians were unable to assign any cause.

**Table 2.** Direct causes of neonatal deaths (n=365) during 2003-2004 in Matlab, Bangladesh

Cause of death	No.	%	95% CI
Birth asphyxia	164	44.9	39.8-50.2
Low birthweight/prematurity	55	15.1	11.6-19.2
Meningitis/sepsis	45	12.3	9.1-16.1
Respiratory distress syndrome	25	6.9	4.5-9.9
Pneumonia	20	5.5	3.4-8.3
Others			
Hypothermia	6	1.6	0.6-3.5
Birth injury	4	1.1	0.3-2.8
Sudden infant death	4	1.1	0.3-2.8
Congenital abnormality	3	0.8	0.2-2.4
Other specified*	6	1.6	1.0-4.3
Undetermined†	26	7.1	4.7-10.3
No cause (unspecified)	7	1.9	0.4-3.2
Total	365	100	

*Neonatal aspiration syndrome (n=4), neonatal jaundice (n=1), and neonatal hypoglycaemia (n=1);

†No agreement between physicians;

CI= Confidence interval

### Cause of death by sex of newborn

There were substantial differences in cause of death when gender was considered (Table 3). Overall, the number of male deaths was 200 compared to 165 female deaths; the males accounted for a larger proportion (54.8%) of all deaths. The following causes of death were proportionately less among females than among males: birth asphyxia (37.6% vs 51%), low birthweight/prematurity (14.5% vs 15.5%), and pneumonia (3.6% vs 7.0%) while the following causes were proportionately greater among females: sepsis/meningitis (15.2% vs 10%), respiratory distress syndrome (7.3% vs 6.5%), and other causes (21.8% vs 10%). Overall, the difference in the proportions of death across cause of death by gender was significant (Pearson χ^2^(5)=15.80; p<0.01).

**Table 3.** Direct cause of death according to sex of neonates, Matlab, Bangladesh

Cause of death	Male (n=200)		Female (n=100)		Both (n=365)	
	No.	%	No.	%	No.	%
Birth asphyxia	102	51.0	62	37.6	164	44.9
Prematurity/low birthweight	31	15.5	24	14.5	55	15.1
Meningitis/sepsis	20	10.0	25	15.2	45	12.3
Pneumonia	14	7.0	6	3.6	20	5.5
Respiratory distress syndrome	13	6.5	12	7.3	25	6.8
Others	20	10.0	36	21.8	56	15.3

Pearson χ^2^(5)=15.80; p<0.01

### Cause of death by early and late neonatal periods

There were major differences in the distribution of causes of death (Table 4) by early and late neonatal periods and across categories of cause of death. These differences were significant [(Pearson χ^2^(5)=130.95; p<0.01; Fisher's Exact p=0.001)]. Birth asphyxia (52.8%) was the single largest category of cause of death in the early neonatal period while sepsis/meningitis (48.3%) was the single largest category in the late neonatal period.

**Table 4.** Direct cause of death according to early and late neonatal period, Matlab, Bangladesh

Cause of death	Early (0-7 days) (n=307)		Late (8-28 days) (n=58)		Both periods (n=365)	
	No.	%	No.	%	No.	%
Birth asphyxia	162	52.8	2	3.4	164	44.9
Prematurity/low birthweight	52	16.9	3	5.2	55	15.1
Respiratory distress syndrome	24	7.8	1	1.7	25	6.8
Meningitis/sepsis	17	5.5	28	48.3	45	12.3
Pneumonia	9	2.9	11	19.0	20	5.5
Others	43	14.0	13	22.4	56	15.3
Specified (rare)	17	5.5	6	10.3	23	6.3
Undetermined	20	6.5	6	10.3	26	7.1
No cause	6	1.9	1	1.7	7	1.9

Fisher's Exact p=0.001

## DISCUSSION

### Neonatal mortality rate

The neonatal mortality rate of 32.3 per 1,000 livebirths during 2003-2004 in Matlab was lower than the national neonatal mortality rate of 41 per 1,000 livebirths and approximated the global average of 33 (2,3). What was particularly encouraging was that this figure represented a 21% reduction in the neonatal mortality rate between 1996 and 2004 in the same area (12), largely as a result of the high coverage of maternal and child-health (MCH) outreach services, albeit with a relatively low rate of skilled/instrumental delivery. The low neonatal mortality rate in the MCH-FP project area in our study compared to the government area may also have been due to the relatively higher use of MCH outreach services and primary-care facilities, including basic obstetric care facilities in the former (13).

### Causes of neonatal death

The proportional distribution of the major causes of neonatal deaths in our study differed somewhat from the WHO-derived global estimates, with important public-health implications. Birth asphyxia accounted for a far larger proportion (45%) of neonatal deaths in Matlab compared to the global average of 23-29% (2,5). The recent survey in Bangladesh referred earlier also showed a higher proportion (39%) of deaths due to this cause (14). Another nationwide perinatal survey in South Africa, conducted during 1999-2003, also reported that asphyxia-hypoxia was responsible for about one-third of neonatal deaths (15). This higher rate of deaths due to birth asphyxia in our study in Bangladesh and in similar developing countries reflects the lack of appropriate resuscitation care for newborns at birth where the great majority of deliveries are conducted in the home with no skilled birth attendance. Also, the high proportion of births that are low weight/premature in Matlab would have contributed to a higher incidence of birth asphyxia as shown in an earlier study in the country (16).

Yet another factor in the case of neonatal fatalities occurring at health facilities is in relation to the inappropriate use of intravenous medications for augmenting the progress of labour. A recent study in India found that unnecessary administration of oxytocics to augment labour was associated with a three-fold increase [odds ratio (OR)=2.6; 95% confidence interval (CI) 1.9-3.6] in birth asphyxia-related deaths (17). An earlier study in Bangladesh also indicated that the trial of labour was associated with an increased risk of neonatal death in health facilities in a rural subdistrict of Bangladesh (18). Results of a recent study in South Africa indicate that inadequate foetal monitoring by health workers is an important avoidable factor associated with birth asphyxia-related deaths (15).

### Premature birth/low birthweight

Prematurity/low birthweight was a cause of death in 15% of the neonatal deaths in our study, which is much lower than the global average of around 27% of neonatal deaths (2,5). It is also lower than those obtained from recent studies in India (19) and Bangladesh (14) where 27% of neonatal deaths were attributed to low birthweight/prematurity. This could have been due to the use of a separate category of respiratory distress syndrome as a cause of death that is usually also associated with prematurity, and this would have left a fewer cases to be assigned to low birthweight/prematurity. Also, it cannot be ruled out that the physicians may have exercised a higher subjective threshold for picking up low-birthweight and premature babies as a cause of death

### Infections

Nearly one-fifth (20.5%) of the deaths in our study were due to infectious diseases, such as sepsis, meningitis, and pneumonia, which underscores the general decline in deaths due to infectious diseases in the country, from a high of 48.9% of all neonatal deaths observed in 1992-1993 in rural Bangladesh (20) and which can be attributed to almost total elimination of tetanus since the introduction of prophylaxis for maternal tetanus. This remains an area for potential intervention to reduce the number of neonatal deaths.

### Unspecified cause

The physicians in our study failed to assign a cause (unspecified) in 1.9% of the deaths, and for another 7.1% of the cases, the cause was listed as undetermined, as they differed on a cause of death. The unspecified proportion (1.9%) is consistent with the latest nationwide neonatal VA survey, which reported 3.4% of neonatal death as unspecified (3). The nationwide VA survey and this study used a similar modular VA tool in collecting information before neonatal death. The use of the new VA tool with a combination of structured questions and open-ended description of symptoms and events yields greater information, including information on healthcare-seeking which enables physicians to allocate a cause for a higher proportion of neonatal deaths. Also, the option to choose a cause of death from an almost unrestricted pool of codes within the ICD-10 classification may have enabled physicians to specify a cause for a greater proportion of neonatal deaths.

While physician's review was considered to be the reference standard in this study, it should be appreciated that assigning the cause of death by the physicians was a largely subjective process, despite the steps taken by the study to ensure uniformity and standardization of the physician's review process.

### Cause of death by sex

The study found that the female neonates had a lower risk of dying than the male ones. Although the study did not assess the factors responsible for such differences, this difference, which is universally reported, is due to biological advantages enjoyed by female infants and the increased vulnerability of male infants in their early life to environmental stresses (21). The explanation of these differentials in cause of death between the sexes observed in this study goes beyond the scope of the study.

### Cause of death by early and late neonatal period

The clustering of deaths found in our study around the early neonatal period mainly due to birth asphyxia and prematurity/low birthweight has been reported from many developing countries (2). This highlights the need for a more focused newborn-care strategy where resuscitation care for birth asphyxia and appropriate management of premature and low-birthweight babies can be made universally available at the household level in developing countries, where deliveries in the home tend to be the norm. In this regard, the use of community health workers to provide home-based care for both birth asphyxia and small size at birth is worth considering (17,22).

### Targeted interventions

A significant proportion of neonatal deaths could be avoided by appropriate resuscitation care. Most asphyxiated neonates can be successfully resuscitated by simple cost-effective measures, such as clearing the airways, drying and stimulating baby by rubbing with a towel to make the baby crying, and, in some cases, by positive pressure ventilation either through mouth-to-mouth breathing or through use of bag and mask (23). Studies in Indonesia and India have demonstrated the effectiveness of using teams of village health workers and traditional birth attendants in reducing the number of deaths due to asphyxia at the community level (17,24).

There is a range of evidence-based interventions available that can improve the survival of premature/low-birthweight babies. The promotion of early and exclusive breastfeeding (25,26), prevention and treatment of hypothermia, including kangaroo mother care (26–28), topical skin-cleansing with chlorhexidine (30), and topical emollient (sunflower oil) treatment for hospitalized infants (31) may reduce morbidity and mortality among low-birthweight and premature infants. Result of a recent community-based study in India provides further evidence that home-based management of low-birthweight and preterm neonates with supportive care and treatment of infections is feasible and effective in improving the survival of newborns (22).

Although infectious causes (sepsis/meningitis and pneumonia) were the major contributor to late neonatal deaths in our study, these are also important in early neonatal deaths. Most early infections are usually acquired from the mother, and management of maternal reproductive and urinary tract infections can, thus, be effective in preventing infections in neonates (32). In addition, community-based studies in developing countries have reported that clean delivery practices (hand-washing and cleaning birth-passages with chlorhexidine) and neonatal care practice just after delivery (clean cord-cutting, applying chlorhexidine over the cord, and skin-cleansing) can also reduce neonatal infections and infection-related mortality (25,30,31). Late neonatal deaths due to neonatal tetanus can be successfully prevented by tetanus toxoid immunization of the pregnant mother and through cleanliness in cord-care (25,30). Although the WHO advocates that very severe neonatal illnesses, including sepsis, be treated in a hospital (33,34), community-based management of sepsis using trained village health workers has been shown to be effective in reducing neonatal mortality (25,35,36).

In summary, for Matlab or similar settings, an integrated package of targeted neonatal interventions for birth asphyxia, prematurity, low birthweight, and infections is key to substantially reducing neonatal mortality. The recommendations for reducing the number of neonatal deaths discussed above require not just health/technical intervention but political commitment to make neonatal and child health a greater priority. Socioeconomic and developmental factors limit the access to and availability of health/technical interventions. Therefore, substantial reduction in neonatal mortality requires health programmes combined with other socioeconomic development activities for the population that facilitate individual and community control over factors determining health.

## ACKNOWLEDGEMENTS

The authors acknowledge the contribution of the study participations. This study was funded by ICDDR,B and its donors which provide unrestricted support to the Centre for its operations and research. Current donors providing unrestricted support include: Australian Agency for International Development (AusAID), Government of the People's Republic of Bangladesh, Canadian International Development Agency (CIDA), Embassy of the Kingdom of the Netherlands (EKN), Swedish International Development Cooperation Agency (Sida), and Department for International Development (DFID), UK. The authors gratefully acknowledge these donors for their support and commitment to the Centre's research efforts. The principal investigator was supported by ICDDR,B and International Postgraduate Research Scholarship (IPRS), Government of Australia.
